# Supplementary material for: Genetic network shaping Kenyon cell identity and function in Drosophila mushroom bodies
Source: eLife. 2026 Feb 27;14:RP108173. doi: 10.7554/eLife.108173 (PMC12948353; doi:10.7554/eLife.108173)
Supplement: Supplementary file 1. [file elife-108173-supp1.docx]

**Supplementary File 1. Expression patterns of GFP lines in Figure 1-S1**

| panel | gene | stock # | in KCs at WL | in KCs at adult |
| --- | --- | --- | --- | --- |
| A1 | *dan* | BDSC92324 | no | no |
| A2 | *dlp* | BDSC60540 | calyx and lobe of KCs | enriched in calyx and lobe of γ |
| A3 | *ed* | BDSC59777 | no | no |
| A4 | *Imp* | DGGR115455 | no | cytosol of γ and α'/β' |
| A5 | *SIFaR* | BDSC60228 | no | no |
| A6 | *TkR86C* | BDSC60549 | no | no |
| B1 | *CG31637* | BDSC64438 | no | no |
| B2 | *CG43373* | BDSC60239 | no | no |
| B3 | *CG4404* | BDSC90835 | no | no |
| B4 | *crb* | BDSC61781 | no | no |
| B5 | *Lmpt* | BDSC66776 | no | no |
| B6 | *mbc* | DGGR115505 | no | no |
| B7 | *Octβ3R* | BDSC60245 | no | no |
| C1 | *Ace* | BDSC60260 | calyx of KCs | calyx of KCs |
| C2 | *app* | BDSC60283 | no | no |
| C3 | *beat-IV* | BDSC66506 | KCs | γ and α'/β' |
| C4 | *Ccn* | BDSC60259 | no | no |
| C5 | *CG4829* | DGGR115623 | no | enriched in α'/β' |
| C6 | *Cyp4p3* | BDSC59829 | no | cytosol and calyx of KCs |
| C7 | *DAT* | VDRC318840 | MB lobe (maybe not KCs) | MB lobe (maybe not KCs) |
| C8 | *dnr1* | BDSC76236 | no | no |
| C9 | *dpr17* | BDSC61801 | no | no |
| C10 | *Epac* | BDSC66364 | no | no |
| C11 | *eys* | BDSC63162 | no | no |
| C12 | *fz3* | VDRC318166 | no | no |
| C13 | *igl* | BDSC60527 | KCs | KCs |
| C14 | *LRP1* | BDSC60248 | no | no |
| C15 | *mamo* | VDRC318601 | no | no |
| C16 | *Mp* | BDSC60567 | no | cytosol and calyx of KCs |
| C17 | *msi* | BDSC61750 | no | enriched in cell body of α'/β' |
| C18 | *Ndae1* | BDSC61778 | no | no |
| C19 | *nuf* | BDSC61802 | no | no |
| C20 | *rhea* | BDSC39649 | no | no |
| C21 | *smal* | VDRC318203 | no | no |
| C22 | *tok* | BDSC60550 | no | no |
| C23 | *Zasp67* | VDRC318355 | no | no |
