# Supplementary material for: Genetic network shaping Kenyon cell identity and function in Drosophila mushroom bodies
Source: eLife. 2026 Feb 27;14:RP108173. doi: 10.7554/eLife.108173 (PMC12948353; doi:10.7554/eLife.108173)
Supplement: Supplementary file 2. [file elife-108173-supp2.docx]

**Supplementary File 2. Genotypes of flies shown in each figure panel**

| **Figure** | **Genotype** |
| --- | --- |
| 1A,1B | *w;+;ab-GFP+;+* |
| 1C,1D,1S3A | *w;Lac-FSVS/+;+;+* |
| 1E,1F | *yw;+;E93-GFSTF/+;+* |
| 1G,1H | *yw,Ca-α1T-GFSTF/+;+;+;+* |
| 2A,3A,4I | *yw,Ca-α1T-GFSTF/w;+;UAS-mCD8::RFP/+;GAL4-OK107/+* |
| 2B | *yw,Ca-α1T-GFSTF/w;UAS-E93-RNAi^BDSC57868^/+;UAS-mCD8::RFP/+;GAL4-OK107/+* |
| 2C | *w;44E04-LexA::P65/+;UAS-mCD8::RFP,lexAop-myr::GFP/+;GAL4-OK107/+* |
| 2D | *w;44E04-LexA::P65/UAS-E93-RNAi^BDSC57868^;UAS-mCD8::RFP,lexAop-myr::GFP/+;GAL4-OK107/+* |
| 2E | *w;70F05-LexA::P65/+;UAS-mCD8::RFP,lexAop-myr::GFP/+;GAL4-OK107/+* |
| 2F | *w;70F05-LexA::P65/UAS-E93-RNAi^BDSC57868^;UAS-mCD8::RFP,lexAop-myr::GFP/+;GAL4-OK107/+* |
| 2G | *w;70F05-LexA::P65/+;lexAop-mCD8::RFP/Ab-GFP;GAL4-OK107/+* |
| 2H | *w;70F05-LexA::P65/UAS-E93-RNAi^BDSC57868^;lexAop-mCD8::RFP/Ab-GFP;GAL4-OK107/+* |
| 2I(1),2S5E(1) | *yw,UAS-Ca-α1T RNAi^BDSC39029^/+;+;+;+* |
| 2I(2), 2S5D(2) | *yw,13F02-AD/UAS-Ca-α1T RNAi^BDSC39029^;70F05-DBD/+;+* |
| 2I(3) | *yw,13F02-AD/+;70F05-DBD/+;+* |
| 2I(4),2S5E(4) | *yw,UAS-E93-RNAi^BDSC57868^/+;+;+;+* |
| 2I(5),2S5C(1),2S5D(3) | *yw,13F02-AD/UAS-E93-RNAi^BDSC57868^;70F05-DBD/+;+* |
| 2I(6),2S5D(1),2S5E(6) | *yw,+;+;+* |
| 3B | *yw,Ca-α1T-GFSTF/w;+;UAS-mCD8::RFP/E93(EP);GAL4-OK107/+* |
| 3C | *w;GAL4-201Y,70F05-LexA::P65/+;UAS-mCD8::RFP,lexAop-myr::GFP/+;+* |
| 3D | *w;GAL4-201Y,70F05-LexA::P65/+;UAS-mCD8::RFP,lexAop-myr::GFP/E93(EP);+* |
| 3E | *w;UAS-mCD8::RFP/+;ab-GFP/+;GAL4-OK107/+* |
| 3F | *w;UAS-mCD8::RFP/+;ab-GFP/E93(EP);GAL4-OK107/+* |
| 3G | *w,mamo^H/I^-HA/w;+;UAS-mCD8::GFP/+;GAL4-OK107/+* |
| 3H | *w,mamo^H/I^-HA/w;+;UAS-mCD8::GFP/E93(EP);GAL4-OK107/+* |
| 3I | *w,mamo^D~G^-HA/w;+;UAS-mCD8::GFP/+;GAL4-OK107/+* |
| 3J | *w,mamo^D~G^-HA/w;+;UAS-mCD8::GFP/E93(EP);GAL4-OK107/+* |
| 3K,2S3A | *w;Lac-FSVS/+;UAS-mCD8::RFP/+;GAL4-OK107/+* |
| 3L | *w;Lac-SVS/+;UAS-mCD8::RFP/E93(EP);GAL4-OK107/+* |
| 4A | *w;+;UAS-mCD8::RFP/Ab-GFP;GAL4-OK107/+* |
| 4B | *w;UAS-E93-RNAi^BDSC57868^/+;UAS-mCD8::RFP/Ab-GFP;GAL4-OK107/+* |
| 4C,4F,1S3B, 2S1A | *yw/w;UAS-mCD8::RFP/+;E93-GFSTF/+;GAL4-OK107/+* |
| 4D | *yw/w;UAS-mCD8::RFP/UAS-chinmo-RNAi;E93-GFSTF/+;GAL4-OK107/+* |
| 4E,4G | *yw/w;UAS-mCD8::RFP/UAS-mamo-RNAi;E93-GFSTF/+;GAL4-OK107/+* |
| 4H | *yw/w;UAS-mCD8::RFP/+;E93-GFSTF/UAS-ab ^BDSC23639^;GAL4-OK107/+* |
| 4J | *yw,Ca-α1T-GFSTF/w;+;UAS-mCD8::RFP/UAS-ab ^BDSC23639^;GAL4-OK107/+* |
| 1S1 | The genotype can be found by stock number listed in Supplementary Table 2 |
| 1S2 | *hs-FLP^[122]^/w;chinmo^1^,FRT^40A^/tubP-GAL80,FRT^40A^;Ab-GFP/UAS-mCD8::RFP;GAL4-OK107/+* |
| 2S1B | *yw/w;UAS-mCD8::RFP/UAS-E93-RNAi^BDSC57868^;E93-GFSTF/+;GAL4-OK107/+* |
| 2S1C | *yw/w;UAS-mCD8::RFP/UAS-E93-RNAi^VDRC104390^;E93-GFSTF/+;GAL4-OK107/+* |
| 2S2A | *yw,Ca-α1T-GFSTF/hs-FLP^[122]^;UAS-mCD8::RFP/+;FRT^82B^/FRT^82B^,tubP-GAL80;GAL4-OK107/+* |
| 2S2B | *yw,Ca-α1T-GFSTF/hs-FLP^[122]^;UAS-mCD8::RFP/+;FRT^82B^,E93^Δ11^/FRT^82B^,tubP-GAL80;GAL4-OK107/+* |
| 2S3B | *w;Lac-SVS/UAS-E93-RNAi^BDSC57868^;UAS-mCD8::RFP/+;GAL4-OK107/+* |
| 2S5E(2) | *yw,GAL4-c739/UAS-Ca-α1T RNAi^BDSC39029^;+;+* |
| 2S5E(3) | *yw,GAL4-c739/+;+;+* |
| 2S5C(2),  2S5E(5) | *yw,GAL4-c739/UAS-E93-RNAi^BDSC57868^;+;+* |
| 3S1B | *w;UAS-mCD8::RFP/UAS-E93-A;ab-GFP/+;GAL4-OK107/+* |
| 3S1C | *w;UAS-mCD8::RFP/UAS-E93-B;ab-GFP/+;GAL4-OK107/+* |
| 3S2A,3S2B | *w;wor-GAL4,UAS-mCD8::RFP/+;ab-GFP/E93(EP);+* |
| 4S1 | *hs-FLP^[122]^/w;chinmo^1^,FRT^40A^/tubP-GAL80,FRT^40A^;E93-GFSTF/UAS-mCD8::RFP;GAL4-OK107/+* |
| 4S2A | *w;UAS-mCD8::RFP/+;E93-GFSTF/UAS-LUC-let7;GAL4-OK107/+* |
| 4S2B | *w;UAS-mCD8::RFP/UAS-syp-RNAi^VDRC33011^;E93-GFSTF/+;GAL4-OK107/+* |
| 4S3 | *yw/w;UAS-mCD8::RFP/+;E93-GFSTF/UAS-ab^F000705^;GAL4-OK107/+* |
| 4S4A,4S5B | *w;UAS-mCD8::RFP/UAS-ab-RNAi^VDRC104582^;ab-GFP/UAS-Dcr2.0;GAL4-OK107/+* |
| 4S4B | *w;UAS-mCD8::RFP/UAS-ab-RNAi^VDRC104582^;E93-GFSTF/UAS-Dcr2.0;GAL4-OK107/+* |
| 4S4C | *yw,Ca-α1T-GFSTF/w;UAS-mCD8::RFP/UAS-ab-RNAi^VDRC104582^;UAS-Dcr2.0/+;GAL4-OK107/+* |
| 4S5A | *w;UAS-mCD8::RFP/UAS-ab-RNAi^VDRC104582^;ab-GFP/+;GAL4-OK107/+* |
| 4S5C | *w;70F05-LexA/UAS-E93-RNAi^BDSC57868^,UAS-ab-RNAi^VDRC104582^;lexAop-mCD8::RFP/ab-GFP;GAL4-OK107/+* |
| 4S5D | *yw,Ca-α1T-GFSTF/UAS-E93-RNAi^BDSC57868^,UAS-ab-RNAi^VDRC104582^;UAS-mCD8::RFP/ UAS-Dcr2.0;GAL4-OK107/+* |
